# Neutralizing antibodies and T-cell responses to inactivated SARS-CoV-2 vaccine in COVID-19 convalescents one and a half years after infection

**DOI:** 10.1016/j.virusres.2022.198977

**Published:** 2022-10-22

**Authors:** Li-Na Yan, Dan Li, Zhen-Dong Wang, Ze-Zheng Jiang, Xiao Xiao, Xue-Jie Yu

**Affiliations:** a State Key Laboratory of Virology, School of Public Health, Wuhan University, Wuhan 430070, PR China; b School of Public Health, Xi'an Medical University, Xi'an 710021, PR China; c Institute of Epidemic Research, Hubei University of Chinese Medicine, Wuhan 430065, PR China

**Keywords:** COVID-19, Neutralizing antibody, T-cell response, Inactivated vaccine, Prior infection, Convalescent

## Abstract

Vaccines have been considered the most promising solution for ending the coronavirus disease 2019 (COVID-19) pandemic. Information regarding neutralizing antibodies (NAbs) and T-cell immune response in inactivated SARS-CoV-2 vaccine-immunized COVID-19 convalescent patients were either only available for a short time after illness recovered or not available at all (T-cell immunity). We evaluated SARS-CoV-2 NAbs and cellular immune responses to the SARS-CoV-2 inactivated vaccine in convalescent patients who recovered from infection for about one and a half years. We found that compared to before vaccination, SARS-CoV-2 NAbs and specific T-cell responses were significantly boosted by the inactivated vaccine in convalescent patients, which confirmed the pre-existing adaptive immunity in SARS-CoV-2 infected people. We observed that NAbs and IFN-γ-secreting T-cell response elicited by a single vaccine dose in subjects with prior COVID-19 infection were higher than after two doses of vaccine in SARS-CoV-2 naïve subjects. Both humoral and cellular immune responses elicited by one and two doses of inactivated vaccine were comparable in COVID-19-recovered persons. In conclusion, inactivated COVID-19 vaccine induced robust NAbs and T-cell responses to SARS-CoV-2 in COVID-19 convalescent patients and immune responses after one dose were equal to that after receiving two doses, which highlighted that robust humoral and cellular immune response can be reactivated by the inactivated vaccine in SARS-CoV-2 convalescent patients.

## Highlights

• SARS-CoV-2 neutralizing antibodies (NAbs) and specific T-cell responses were significantly boosted by the inactivated vaccine in convalescent patients 17–18 months after illness onset.
• NAbs and IFN-γ-secreting T-cell response elicited by a single vaccine dose in subjects with prior COVID-19 infection were higher than the levels in uninfected people who had received two doses of the vaccine.
• Both humoral and cellular immune responses elicited by one and two doses of inactivated vaccine were comparable in COVID-19-recovered persons.

## 1 Introduction

The coronavirus disease 2019 (COVID-19) pandemic caused an unprecedented crisis and threat in a century. As of October 11, 2022, more than 618 million COVID-19 cases had been confirmed worldwide, with more than 6.5 million confirmed deaths (World Health Organization, 2022a). Mass vaccination is considered the most promising approach to ending the COVID-19 global health threat. Up to now, approved COVID-19 inactivated vaccines require a first dose followed 14 to 28 days later by a second dose (Xia et al., 2020;Xia et al., 2021)). In many countries and regions, vaccinations were carried out on all volunteer subjects regardless of the history of SARS-CoV-2 infection. There are two fundamental issues in the vaccination of COVID-19 convalescent patients. First, is it necessary to vaccinate those who had a history of COVID-19? Second, is it necessary to administer the second dose of the vaccine?

Several studies reported humoral immunity to a single or two doses of messenger RNA (mRNA) vaccines and adenovirus-vectored vaccines in subjects previously infected with SARS-CoV-2 (Chong et al., 2021; Ebinger et al., 2021; Goel et al., 2021; Havervall et al., 2021; Krammer et al., 2021; Manisty et al., 2021; Reynolds et al., 2021; Saadat et al., 2021; Lim et al., 2022). These COVID-19 convalescents developed strong spike-specific IgG and neutralizing antibodies (NAbs) after one dose that were equivalent to or exceeding those naïve recipients after two-dose vaccination (Ebinger et al., 2021; Goel et al., 2021; Havervall et al., 2021; Krammer et al., 2021; Manisty et al., 2021; Reynolds et al., 2021; Saadat et al., 2021). Current World Health Organization guidelines recommend a two-dose inactivated vaccine regardless of a person's history of symptomatic or asymptomatic SARS-CoV-2 infection (World Health Organization, 2022b). Data on immune responses of the inactivated vaccine in individuals who had infected with SARS-CoV-2 before vaccination was limited. Especially, we do not know the effectiveness of the inactivated vaccine in COVID-19 convalescents who recovered from infection longer than one year. In this study, we evaluated NAbs and T-cell response to SARS-CoV-2 (wild-type strain) in individuals vaccinated with one or two doses of SARS-CoV-2 inactivated vaccines after recovering from COVID-19.

## 2 Methods

### 2.1 Study population

**Fig. 1:**
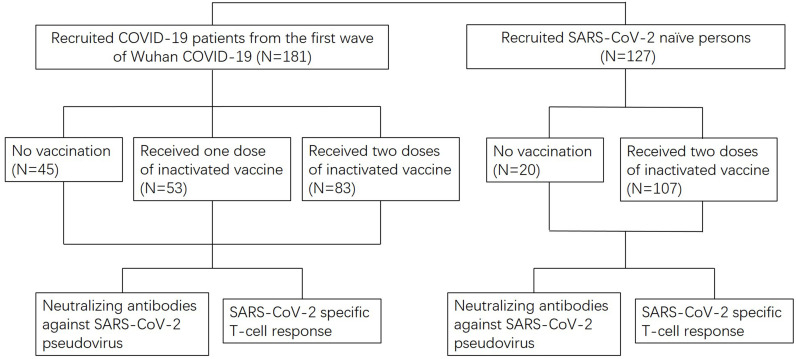
Study schema. A total of 308 individuals were enrolled in this study including 181 COVID-19 convalescent patients 17–18 months after illness onset and 127 SARS-CoV-2 naïve persons. All vaccinees were sampled within two months after the last vaccination. A total of 20 unvaccinated healthy persons were recruited as negative controls.

The study was approved by the Ethics Committee of Wuhan University. All participants wrote informed consent. Inclusion criteria were as follows: (1) with or without a history of COVID-19; (2) received one or two doses of SARS-CoV-2 inactivated vaccines. A total of 308 persons were recruited in this study, including 181 COVID-19 convalescent patients and 127 persons who were never infected (SARS-CoV-2 naïve). All convalescent patients were diagnosed with COVID-19 and were hospitalized due to COVID-19 in the first wave of COVID-19 in Wuhan between December 30, 2019, and February 24, 2020 (Fig. 1). Blood samples were collected from all participants 17–18 months after COVID-19 onset. All vaccinees were sampled within two months after the last vaccination. All sera were heat-inactivated and stored at -80 °C. Peripheral blood mononuclear cells (PBMCs) were isolated from fresh blood and used immediately after isolation.

### 2.2 SARS-CoV-2 pseudovirus production

NAbs and T-cell immune response to SARS-CoV-2 wild-type strain (WT) isolated initially from Wuhan were evaluated. SARS-CoV-2 WT spike plasmid (GenBank: MN908947) was constructed by us previously (Yan et al., 2022). HEK293T cells were cotransfected with pNL4.3-Luc.R-E- and SARS-CoV-2 WT spike plasmid (ratio 3:1). Cell supernatants containing pseudovirus were harvested 48 h (h) after transfection and pseudoviruses were titered with human angiotensin-converting enzyme 2 (ACE2) overexpressed Vero-E6 cells (Vero-E6_ACE2_).

### 2.3 SARS-CoV-2 neutralizing antibodies

Vero-E6_ACE2_ cells were seeded in 96-well plates (NEST Biotechnology, Wuxi, China) to a density of 2 × 10^4^ cells/well. Serum samples were two-fold diluted starting from 1:8. SARS-CoV-2 pseudoviruses (5 × 10^4^ relative luminescence units, RLU) were mixed with an equal volume of diluted sera and incubated for 1 h at 37 °C. Then, the mixture of a patient's serum and SARS-CoV-2 pseudoviruses was added to a well of a 96-well plate for 72 h. Luciferase activity was tested using Bio-Lite Luciferase Assay Kit (Vazyme Biotech co., Ltd, Nanjing, China) and Tecan Spark multifunction microplate reader (Tecan, Austria). The neutralizing antibody titer was defined as the maximum dilution of serum at 50% neutralization (ND_50_). The positive threshold of this assay was 1:8.

### 2.4 ELISpot assay

SARS-CoV-2 WT spike peptide pools (15 mers 11 overlap) (313 peptides, 1-1267 amino acid) (SinoBiological, Beijing, China) were used as specific stimulation. SARS-CoV-2 T-cell response was quantified with ELISpot assays using Human IFN-γ and IL-2 ELISpot Kits (Dakewe Biotech, Shenzhen, China). In brief, PBMCs were isolated from the blood of COVID-19 patients and healthy persons. A total of 5 × 10^5^ PBMCs were seeded in pre-coated wells and stimulated with spike peptide pools (2 μg/ml) for 24 h. Phytohemagglutinin (PHA, 2.5 μg/ml) and dimethyl sulfoxide (DMSO) were used as the positive and negative controls, respectively. Spots were counted with an ELISpot reader (Mabtech, Stockholm, Sweden). A positive reaction was defined as three times above the mean spots of the negative controls.

### 2.5 Statistical analysis

The  comparison of categorical data was conducted using χ^2^ test and Fisher exact test. The Mann-Whitney test was used to evaluate differences in immune responses between groups. Spearman rank correlation test was used to evaluate the correlation between NAbs and T-cell response. SPSS 22.0 software and GraphPad Prism 8.3.0 were used to analyze and draw. *P* < 0.05 was considered statistically significant. *, *P* < 0.05; **, *P* < 0.01; ***, *P* < 0.001.

## 3 Results

### 3.1 Demographic characteristics

We recruited 181 COVID-19 convalescent patients and 127 SARS-CoV-2 naïve persons (Fig. 1). These 181 COVID-19 patients were from the first wave of COVID-19 in Wuhan. The blood sampling time was from July 13, 2021, to July 28, 2021. These convalescent patients recovered about 17 to 18 months ago from COVID-19 onset. The median age of the patients was 56 years (IQR, 49-65 years). Among them, 103 (56.9%) were younger than 60 and 78 (43.1%) were ≥60 years old. 145 (80.1%) patients had mild clinical manifestations and 36 (19.9%) were in severe condition, and 68 (45.6%) patients were female. For those 107 vaccinated SARS-CoV-2 naïve persons, the sampling time was 26–31 days after the last vaccination. The information of persons enrolled in this study was listed in Table 1, including sex, age, disease severity, times after symptom onset to blood sampling, and the days after the last COVID-19 vaccine.

**Table 1:** The information of enrolled participants.

	Total (*N* = 308)	COVID-19 convalescent			*P* value	COVID-19 naïve (*N* = 107)		*P* value
		no vaccine (*N* = 45)	one dose (*N* = 53)	two doses (*N* = 83)		no vaccine (*N* = 20)	two doses (*N* = 107)	
Sex								
Male	120 (39.0)	25 (55.6)	23 (43.4)	34 (41.0)	0.27^a^	7 (35.0)	31 (29.0)	0.589^a^
Female	188 (61.0)	20 (44.4)	30 (56.6)	49 (59.0)		13 (65.0)	76 (71.0)	
Age,year,median (IQR)	49 (33,61.75)	54 (48,63)	58 (52,66)	56 (48,65)		38 (27,42)	29 (24,36)	
Age (year)								
<60, n (%)	224 (72.7)	28 (62.2)	27 (50.9)	48 (57.8)	0.518^a^	18 (90.0)	103 (96.3)	0.239^b^
≥60, n (%)	84 (27.3)	17 (37.8)	26 (49.1)	35 (42.2)		2 (10.0)	4 (3.7)	
Severity of COVID-19, n (%)								
Mild	145 (80.1)	36 (80.0)	43 (81.1)	66 (79.5)	0.974^a^	NA	NA	
Severe	36 (19.9)	9 (20.0)	10 (18.9)	17 (20.5)		NA	NA	
Days after the last SARS-CoV-2 vaccination, Median (IQR)	NA	NA	26 (14,48)	31 (24,50)	0.145^a^	NA	29 (21,44)	
Internals of two doses of vaccine, days, Median (IQR)	NA	NA	NA	29 (24,50)		NA	35 (22,50)	
Times after COVID-19 onset	17-18 months					NA	NA	

Abbreviations: n, number; IQR, IQR, interquartile range; NA, not available.

a *P* value was calculated using x^2^ test.

b *P* value was calculated using Fisher exact test.

### 3.2 Neutralizing antibodies

**Fig. 2:**
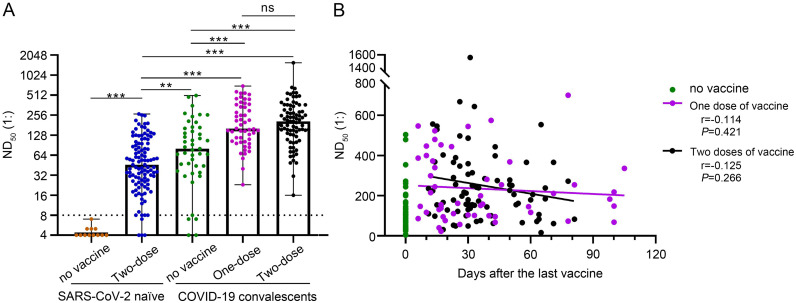
Neutralizing antibodies (ND_50_) after receiving the inactivated SARS-CoV-2 vaccine in COVID-19 convalescents. (A) ND_50_ levels in COVID-19 convalescents and SARS-CoV-2 naïve persons after receiving the inactivated vaccine. The differences in ND_50_ between groups were analyzed using the Mann-Whitney U test. **, *P* < 0.01; ***, *P* < 0.001; ns, not significant. (B) The correlation between ND_50_ and the days after the last vaccination in COVID-19 convalescents. The spearmen rank correlation test was used to evaluate the correlation.

Previously we found that humoral and cellular immunity induced by SARS-CoV-2 natural infection persisted for at least 17–18 months after COVID-19 onset (Yan et al., 2022). To investigate how inactivated SARS-CoV-2 vaccines affect immune responses in COVID-19-recovered people, we tested NAbs against SARS-CoV-2 pseudovirus in individuals vaccinated with the inactivated SARS-CoV-2 vaccine who were SARS-CoV-2 naïve or infected with 17–18 months ago (COVID-19 convalescents). Two months after vaccination, the positive rate of NAbs was 92.5% (99/107) in SARS-CoV-2 naïve group and all the COVID-19 convalescents developed detectable NAbs after receiving the first dose (100%, 53/53), and the second dose (100%, 83/83) of the vaccine. Serum NAbs of COVID-19 convalescents after receiving one or two doses of inactivated SARS-CoV-2 vaccine were significantly higher than those before vaccination (*P* < 0.001 and *P* < 0.001). The NAb titers were not significantly different (*P* = 0.58) between COVID-19 convalescents who received one dose and two doses of vaccine (Fig. 2**A**). After receiving one and two doses of inactivated vaccines, COVID-19 convalescents had significantly higher NAbs (3.2 fold and 2.6 fold) than the levels in uninfected people who had received two doses of the vaccine.

According to our previous studies, NAbs induced by inactivated vaccine tapered from the third month in SARS-CoV-2 naïve subjects (Yan et al., 2022). We conducted a longitudinal study to observe the trend of the NAbs of the COVID-19 convalescents after receiving the first and second vaccines. No significant correlation was found between NAbs and time after the last vaccination in the one-dose group in SARS-CoV-2 convalescent patients (r=-0.114, *P*=0.421). Similar results were observed in the two-dose group (r=-0.125, *P*=0.266) (Fig. 2B). These results indicated that SARS-CoV-2 inactivated vaccine induced a durable neutralizing antibody response in COVID-19 convalescents.

### 3.3 SARS-CoV-2 specific T-cell response

**Fig. 3:**
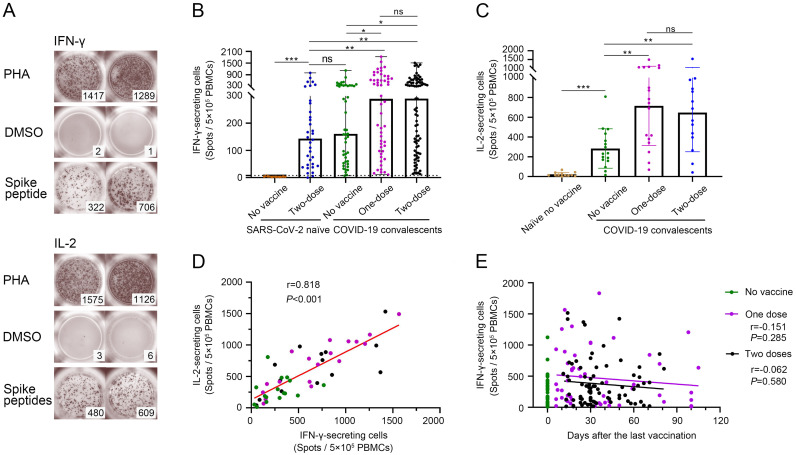
Specific T-cell response to SARS-CoV-2 of COVID-19 convalescents after receiving the inactivated SARS-CoV-2 vaccine. (A) The respective IFN-γ and IL-2 ELISpots from three COVID-19 convalescents against SARS-CoV-2 WT spike peptides, with PHA as the positive control and DMSO as the negative control. (B) IFN-γ-secreting T-cells in COVID-19 convalescents and SARS-CoV-2 naïve persons after receiving the inactivated SARS-CoV-2 vaccine. (C) IL-2-secreting T-cells in COVID-19 convalescents after receiving the inactivated SARS-CoV-2 vaccine. The difference in T-cell responses between groups was analyzed using the Mann-Whitney U test. *, *P* < 0.05; **, *P* < 0.01; ***, *P* < 0.001; ns, not significant. (D) The correlation between IFN-γ-secreting T-cell response and IL-2-secreting T-cell response. (E) The correlation between IFN-γ-secreting T-cell response and the days after the last vaccination in COVID-19 convalescent patients. The spearmen rank correlation test was used to evaluate the correlation.

Using ELISpot assay, we tested SARS-CoV-2 specific IFN-γ and IL-2-secreting T-cell responses in COVID-19 convalescents, respectively. The respective IFN-γ and IL-2 ELISpots from four COVID-19 convalescents against SARS-CoV-2 spike peptides were presented, with PHA as the positive control and DMSO as the negative control (Fig. 3A). We found that 91.2% (31/34) of SARS-CoV-2 naïve persons had IFN-γ-secreting T-cell response within two months after receiving two doses of inactivated vaccines (median: 143.5 spot-forming cells (SFCs)/5 × 10^5^ PBMCs, IQR: 46, 275.5 SFCs/5 × 10^5^ PBMCs). After 17–18 months of natural infection, 88.8% (40/45) of convalescents had SARS-CoV-2 IFN-γ-secreting T-cell response (median: 161 SFCs/5 × 10^5^ PBMCs, IQR: 59.5, 353.5 SFCs/5 × 10^5^ PBMCs). The difference in IFN-γ-secreting T-cell response levels between two-dose vaccinated naïve individuals and COVID-19 convalescents was not significant (*P* = 0.33). (Fig. 3B)

Compared to prior vaccination, IFN-γ-secreting T-cell responses were significantly higher after receiving the first dose and the second dose vaccine (1.6 and 1.9 fold) in convalescent patients, respectively (*P* < 0.001 and *P* < 0.001) (Fig. 3B). Similar results were observed in the IL-2-secreting T-cell response (Fig. 3C). Of great interest, we found that IFN-γ-secreting T-cell responses in COVID-19 convalescents after the first dose were comparable to those after the second dose (median: 101.5 *vs* 95.5, *P* = 0.47) (Fig. 3B). Also, IL-2-secreting T-cell responses in COVID-19 convalescents after the first dose were comparable to those after the second dose (median: 370.5 *vs* 363.5, *P* = 0.54) (Fig. 3C). A strong correlation was found between IFN-γ-secreting T-cell response and IL-2-secreting T-cell response (*r* = 0.8184, *P* < 0.001) (Fig. 3D)

According to our previous studies, T-cell response induced by inactivated vaccine tapered from the third month in SARS-CoV-2 naïve individuals (Yan et al., 2022). We want to explore whether the SARS-CoV-2 specific T-cell response waned when the vaccination time went in COVID-19 convalescents. No significant correlation was found between IFN-γ-secreting T-cell response and vaccination time in the one-dose group in COVID-19 convalescents (r=-0.151, *P*=0.285). Similar results were observed in the two-dose group (r=-0.062, *P*=0.580) (Fig. 3E). These results indicated that inactivated SARS-CoV-2 vaccine induced durable T-cell response in COVID-19 convalescents.

### 3.4 The correlation of immune responses induced by inactivated SARS-CoV-2 vaccine in COVID-19 convalescents

**Fig. 4:**
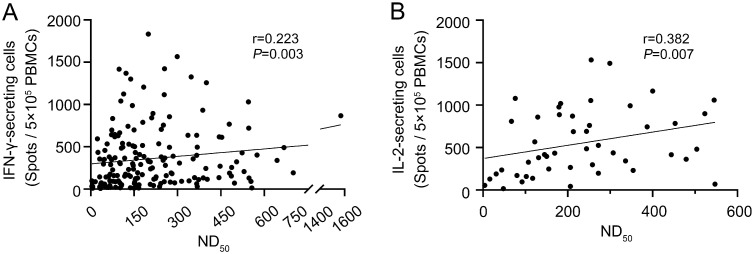
The correlation of immune responses induced by inactivated SARS-CoV-2 vaccine in COVID-19 convalescents. (A) The correlation of IFN-γ-secreting T-cells with neutralizing antibodies (ND_50_) in 181 COVID-19 convalescents. (B) The correlation of IL-2-secreting T-cells with ND_50_ in 181 COVID-19 convalescents. The spearmen rank correlation test was used to evaluate the correlation.

Previous studies have shown that NAbs correlated with T-cell response after SARS-CoV-2 natural infection (Cassaniti et al., 2021; Pan et al., 2021). Little information was available about the relationship between immune responses in COVID-19 convalescents after the booster of the inactivated vaccine. We found that NAbs correlated well with IFN-γ-secreting and IL-2-secreting T-cell responses after receiving the inactivated vaccine in COVID-19 convalescents (*P* < 0.01 and *P* < 0.01) (Fig. 4A and 4B), which indicated a positive correlation between cellular immune and humoral immune response in vaccinated COVID-19 convalescent patients.

## 4 Discussion

Safe and effective vaccines are critical to control the unprecedented COVID-19 pandemic. More than 172 vaccines were in clinical trials (World Health Organization, 2022c). World Health Organization listed eleven COVID-19 vaccines for emergency use (World Health Organization, 2022d). Inactivated vaccine (CoronaVac) was authorized as an emergency using vaccine and widely used in more than 56 countries (COVID-19 Vaccine Tracker, 2022). Inactivated COVID-19 vaccine had been demonstrated good safety and effectiveness in several clinical trials (Xia et al., 2020; Al Kaabi et al., 2021; Jara et al., 2021; Wu et al., 2021).

At present, few researchers have reported the immune responses of patients who recovered from COVID-19 over a year (Choe et al., 2021; Dan et al., 2021; Feng et al., 2021; Peng et al., 2021; Wang et al., 2021; Zhang et al., 2021). Little information was available about the immune responses of the inactivated vaccine in individuals who recovered from COVID-19 over a year. NAbs and antigen-specific memory T-cells had been implicated as critical indexes to evaluate the immunogenicity of vaccines which played a crucial role in controlling viral infection. Previous studies have focused more on the role of antibodies or NAbs while paying less attention to the role of T-cell immunity. Our study focused on both NAbs and virus-specific cellular immune response induced by a booster of the inactivated vaccine in convalescent patients one and a half years after COVID-19 recovery.

Our previous studies suggested that levels of NAbs in SARS-CoV-2 naïve individuals were weak after receiving one dose of inactivated vaccine with a sera-positive rate of 41.2%, and the NAbs increased rapidly after the second dose with a positive rate of over 90%, confirming that the second dose was necessary for SARS-CoV-2 naïve persons (Yan et al., 2022). In this study, we found that COVID-19 convalescents remained a strong neutralizing antibody titer and a robust T-cell response one and a half years after SARS-CoV-2 natural infection, which was higher or equal to that of SARS-CoV-2 naïve people who received two doses of inactivated vaccines. We found that compared to prior vaccination, SARS-CoV-2 specific T-cell responses and NAbs were significantly boosted by the inactivated vaccine in convalescent patients, which confirmed the pre-existing adaptive immunity in SARS-CoV-2 infected people (Karlsson et al., 2020; Noh et al., 2021). We observed that both NAbs and virus-specific T-cell responses in convalecent persons who received one dose of inactivated vaccine were comparable to those in persons who received two doses of vaccine. It indicated that a single dose of inactivated vaccine induced strong NAbs and virus-specific T-cell response and the second booster immunization did not further increase the strength of NAbs or T cell immunity in COVID-19 convalescents. Similar results were found in convalescent patients who received the COVID-19 mRNA vaccine (Chong et al., 2021; Havervall et al., 2021; Krammer et al., 2021; Manisty et al., 2021; Lim et al., 2022). Joseph et al. showed that SARS-CoV-2 spike IgG antibody response after a single dose of BNT162b2 (Pfizer-BioNTech) mRNA vaccine in previously infected individuals was similar to the antibody response seen after two doses in those recovered recipients (Ebinger et al., 2021). In a small cohort, Yong et al. found anti-spike IgG levels in SARS-CoV-2-recovered healthcare workers were comparable after one and two doses of the BNT162b2 mRNA vaccine, and similar results were observed in previously infected nursing home residents (Chong et al., 2021). Our study and previous studies suggested that only a single vaccine dose could induce a strong humoral and cellular immune response in COVID-19 convalescents (Chong et al., 2021; Ebinger et al., 2021).

Previous researchers studied the effects of the vaccine in individuals who experienced SARS-CoV-2 infection 2 to 9 months before vaccination, and little information was available that whether a single dose of vaccine might be effective in people who recovered from infection longer than 9 months before vaccination (Ebinger et al., 2021; Havervall et al., 2021; Wise, 2021; Lim et al., 2022). In this study, we found that vaccine booster immunization was still effective after up to 15–17 months of SARS-CoV-2 infection. Besides, no significant correlation was found between immune response (including NAbs and T-cell response) and the days after vaccination in SARS-CoV-2 convalescent patients, indicating that inactivated vaccine induced prolonged both humoral and cellular immune responses in COVID-19-recovered individuals. We found a positive correlation between NAbs and T-cell response in COVID-19 convalescents after receiving the inactivated vaccine. Individuals with high levels of NAbs tended to have high levels of cellular immunity.

In summary, we found inactivated SARS-CoV-2 vaccine induced robust NAbs and T-cell responses to SARS-CoV-2 in COVID-19 convalescent patients 15–17 months after SARS-CoV-2 infection and immune responses after one dose was equivalent to that after receiving two doses, which highlighted that robust humoral and cellular immune response can be reactivated by the inactivated vaccine in SARS-CoV-2 convalescent patients. How long the NAbs and cellular immune response can last and the immune response against current circulating Omicron variants needs to be explored in the future.

## Funding

This research was supported by the National Natural Science Foundation of China (No. 81971939), the National Natural Science Foundation of China (NSFC) Young Scientist Fund (No. 82104566), and the Research and Development Program of Hubei Province (No. ZY.2021F015).

## CRediT authorship contribution statement

**Li-Na Yan:** Conceptualization, Methodology, Visualization, Software, Resources, Formal analysis, Writing – original draft, Writing – review & editing. **Dan Li:** Resources, Writing – review & editing. **Zhen-Dong Wang:** Formal analysis, Writing – review & editing. **Ze-Zheng Jiang:** Resources, Writing – review & editing. **Xiao Xiao:** Resources, Writing – review & editing. **Xue-Jie Yu:** Conceptualization, Methodology, Visualization, Funding acquisition, Writing – review & editing.

## Declaration of Competing Interest

The authors declare that they have no known competing financial interests or personal relationships that could have appeared to influence the work reported in this paper.

## Data Availability

No data was used for the research described in the article.
